# Greater cerebellar gray matter volume in car drivers: an exploratory voxel-based morphometry study

**DOI:** 10.1038/srep46526

**Published:** 2017-04-18

**Authors:** Hiroyuki Sakai, Takafumi Ando, Norihiro Sadato, Yuji Uchiyama

**Affiliations:** 1 Human Science Research Domain, Toyota Central R&D Laboratories, Inc., Nagakute, Aichi 480-1192, Japan; 2 Division of Cerebral Integration, National Institute of Physiological Sciences, Okazaki, Aichi 444-8585, Japan

## Abstract

Previous functional neuroimaging studies have identified multiple brain areas associated with distinct aspects of car driving in simulated traffic environments. Few studies, however, have examined brain morphology associated with everyday car-driving experience in real traffic. Thus, the aim of the current study was to identify gray matter volume differences between drivers and non-drivers. We collected T1-weighted structural brain images from 73 healthy young adults (36 drivers and 37 non-drivers). We performed a whole-brain voxel-based morphometry analysis to examine between-group differences in regional gray matter volume. Compared with non-drivers, drivers showed significantly greater gray matter volume in the left cerebellar hemisphere, which has been associated with cognitive rather than motor functioning. In contrast, we found no brain areas with significantly greater gray matter volume in non-drivers compared with drivers. Our findings indicate that experience with everyday car driving in real traffic is associated with greater gray matter volume in the left cerebellar hemisphere. This brain area may be involved in abilities that are critical for driving a car, but are not commonly or frequently used during other daily activities.

Driving is a complex everyday activity that requires multiple types of sensory processing, cost-weighted decision making, precise motor control, and other abilities. Even on an empty road, drivers must continuously operate the steering wheel and pedals in consideration of complicated vehicle dynamics. Driving is also a vigilance task, which is often undertaken for prolonged periods of time, and carries a constant risk of injury or death resulting from collisions. Despite this, driving is commonly thought to provide pleasure, at least, in certain circumstances or among car enthusiasts. Each of these features shapes the peculiarities of car driving in everyday life experiences.

It is widely accepted that experience can alter the structure of the brain. Among the different types of experience, musical training-related brain structural plasticity has been most extensively studied (see ref. 1 for review). Converging evidence clearly indicates that musical skill acquisition modifies the structure of motor^2^,^3^,^4^,^5^ and auditory^4^,^5^,^6^ cortices. More recent studies have suggested that intensive and controlled training is not mandatory for experience-induced brain structural plasticity. For instance, brain structural plasticity can be induced by everyday leisure activities, such as golfing^7^ and playing video games^8^,^9^,^10^,^11^,^12^.

Given the above, it is highly likely that everyday car-driving experience modulates the structure of specific brain regions associated with the demands of driving a car in real traffic. However, few studies have investigated brain morphology associated with car-driving experience. Maguire *et al*.^13^ reported that gray matter (GM) volume in the posterior hippocampus of London taxi drivers was greater than that in non-taxi drivers, and that GM volume correlated positively with number of years spent working as a taxi driver. These findings and those of subsequent studies^14^,^15^ strongly suggest that greater hippocampal volume in taxi drivers reflects the acquisition and/or daily use of detailed spatial representation, rather than innate navigation expertise per se. More recently, Bernardi *et al*.^16^ conducted a voxel-based morphometry (VBM) study that revealed that, compared with non-experienced drivers, professional auto racing drivers had greater GM volume in various brain regions including the basal ganglia, sensorimotor cortex, inferior frontal gyrus, retrosplenial cortex, lingual gyrus, and parahippocampus. However, no studies have investigated brain morphology associated with everyday car-driving experience in ordinary drivers.

Thus, in the current study, we investigated differences in GM volume between ordinary drivers and non-drivers. To this end, we recruited university students who had either a few years’ or no driving experience and collected structural brain images for a whole-brain VBM analysis. We chose to study this population to minimize possible confounding variables between drivers and non-drivers (i.e., demographic, cultural, social, and educational factors). Nevertheless, it is possible that physical activity was a confounding group difference. According to a previous study examining the impacts of different travel modes on physical activity in university students^17^, the use of motor vehicles as a driver was associated with reduced physical activity. In addition, individual predispositions characterized by GM volume in specific brain regions^18^,^19^ might determine interest in car driving. To address these possibilities, we also examined between-group differences in habitual physical activity and general personality traits using respective questionnaires.

## Results

### Group characteristics

Descriptive statistics for drivers and non-drivers are summarized in Table 1. As a result of the visual inspection of structural brain images, one driver and two non-drivers were excluded from further analysis. Consequently, 34 drivers and 36 non-drivers were included in the subsequent analysis. The mean age difference between drivers and non-drivers was slight (21.5 ± 0.9 *vs* 21.0 ± 0.8 years) but statistically significant (*P* = 0.033). Meanwhile, there were no between-group differences (*P* > 0.05) in gender, personality traits assessed via the NEO Five-Factor Inventory (NEO-FFI)^20^, and habitual physical activity assessed by the the International Physical Activity Questionnaire (IPAQ)^21^.

### VBM analysis

**Figure 1:**
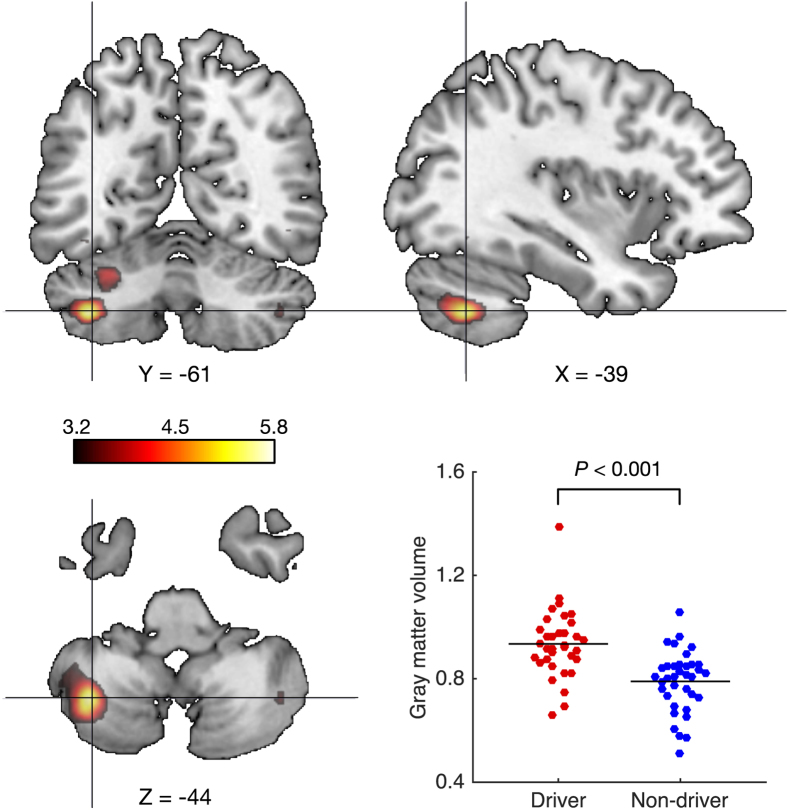
Group differences in GM volume between drivers and non-drivers. The red-colored voxels represent regions showing greater GM volume in drivers compared with non-drivers (uncorrected *P* < 0.001 for visualization purpose only). The voxels in the left cerebellar hemisphere (*x* = −39, *y* = −61, *z* = −44; *k* = 30) survived the multiple comparison correction (family-wise error corrected *P* < 0.05). No brain areas showed a significantly larger GM volume in the opposite contrast (i.e., drivers < non-drivers). The scatter plot compares the mean GM volume in the significant voxels between drivers and non-drivers.

The whole-brain VBM analysis found brain regions showing greater GM volume (adjusted for age) in drivers compared to non-drivers, including the bilateral cerebellum and the bilateral fusiform gyrus (Fig. 1). Among them, the left cerebellar hemisphere survived the multiple comparison correction (family-wise error corrected *P* < 0.05; Table 2 and Fig. 1). In contrast, no brain areas showed a significantly larger GM volume in non-drivers compared with drivers.

## Discussion

The current VBM study demonstrated that, relative to non-drivers, drivers had greater GM volume in the left cerebellar hemisphere. In addition, we were not able to identify any potential confounding factors responsible for the observed structural differences between drivers and non-drivers, i.e., habitual physical activity and general personality traits. Our results suggest that driving experience in real traffic is associated with GM volume in the left cerebellar hemisphere. This brain area may be involved in abilities that are critical for car driving, but are not commonly or frequently used during other daily activities.

Cerebellar activation during car-driving tasks has been frequently reported in previous neuroimaging studies^22^,^23^,^24^,^25^,^26^,^27^,^28^,^29^ (see also ref. 30 for review), although there is large variation in the reported cerebral activation patterns. This is not surprising because driving tasks usually require fine motor control for handling input devices, and the cerebellum is crucial for fine motor control. Thus, the greater cerebellar GM volume found in car drivers might be attributable to driving-related fine motor control. In general, each side of the cerebellum controls the ipsilateral side of the body; that is, the left cerebellar hemisphere is implicated in motor control of the left side of the body, and vice versa. Car driving as a motor task requires coordination of the bilateral hands and feet for steering and pedaling, regardless of their dominance. This unique feature of car driving, i.e., the requirement of fine motor control of the left (non-dominant) limbs, might initiate a left-lateralized cerebellar GM increase in right-handed drivers. If so, the opposite (right) side of the cerebellum would be expected to have greater GM volume in left-handed drivers compared with left-handed non-drivers. Moreover, laterality in traffic conditions (i.e., left-hand traffic and right-hand steering wheel for our study population) might be associated with the observed laterality in cerebellar GM volume in drivers. This will be tested in a replication study in a country with reversed traffic laterality. Future research should investigate these issues in more depth.

However, driving-related cerebellar activation is not necessarily restricted to fine motor control. Extensive bilateral cerebellar activation has also been reported in studies where input devices for the vehicle control were operated with the right hand only^26^,^27^,^28^. In addition, according to a study of human cerebellar sensorimotor representations by Grodd *et al*.^31^, the left cerebellar hemisphere (where we found greater GM volume in drivers) does not seem to overlap with somatotopic maps in the cerebellum. These results enable us to speculate that the left cerebellar hemisphere is responsible for functions involving the non-motor peculiarities of car driving. According to a theoretical framework proposed by Michon^32^, driving skills can be divided into three levels: strategic, tactical, and operational skills. At the strategic level, an important skill is to determine an appropriate balance of risk and cost for safe driving. At the tactical level, drivers must determine the manoeuvring of a vehicle depending on the traffic situation. At the operational level, drivers must execute the determined manoeuvres. In general, operational skills require fine motor control, while tactical and strategic skills may be considered non-motor characteristics of car driving.

One possible non-motor characteristics of car driving that could account for greater cerebellar GM volume in drivers is the acquisition and operation of internal models reflecting complex vehicle dynamics. To maneuver a car as intended, drivers need to operate a steering wheel and pedals while considering the inverse dynamics of both vehicle and limbs. Furthermore, many studies have indicated that drivers execute anticipatory control using a forward model of vehicle dynamics to achieve a desired trajectory^33^,^34^,^35^. The cerebellum is thought to acquire and retain internal inverse and forward models of motor apparatuses (see ref. 36 for review). In addition, Imamizu *et al*.^37^ used a visual tracking task with a mouse device to demonstrate the involvement of the cerebellum in the acquisition and operation of tool internal models. The same research group further demonstrated the modular organization of internal models of tools in the cerebellum^38^,^39^. More recently, Galea *et al*.^40^ found that anodal transcranial direct current stimulation of the cerebellum increased the speed of adaptation to a novel visuomotor transformation during a tool-use task. These data represent evidence of cerebellar involvement in the acquisition and operation of internal models for handling tools.

Another possible non-motor peculiarity of car driving is visual attention. Numerous epidemiological studies have highlighted the remarkable relationship between visual attention capacity and traffic accident risk^41^,^42^,^43^,^44^. Indeed, driving a car is a task that demands a high degree of visual attention. Allen *et al*.^45^ found that activation in the left cerebellar hemisphere was associated with attentional processes, independent of motor involvement. The findings of a lesion study by Gottwald *et al*.^46^ also support the involvement of the cerebellum in attentional processes. Moreover, a functional connectivity study^47^ revealed that the left cerebellar hemisphere projects to the right frontoparietal regions that are considered pivotal for visual attention control^48^,^49^. A recent functional connectivity analysis^50^ also provided evidence for a significant role of the cerebellum in sustained attention. Thus, attention-related cerebellar activation during car driving could be associated with the observed increase in cerebellar GM in drivers. Note, however, that cerebellar involvement in visual attentional processes is still somewhat controversial. For instance, several lesion studies found intact attentional capacity in cerebellar patients^51^,^52^.

Mental manipulation of visual representations might also be an alternative account for the current result. During car driving, for instance, drivers must utilize visual information in the sideview and rearview mirrors by projecting it onto their own egocentric coordinate framework. In addition, goal-directed navigation and map reading skills, which are definitely important cognitive components for car driving, are highly correlated with mental rotation task performance^53^. Such a high demand for the mental manipulation of visuospatial information is likely a cognitive peculiarity of car driving. Jordan *et al*.^54^ showed that cerebellar activation is associated with mental rotation of three-dimensional objects. Further, Stoodley *et al*.^55^ explored the functional topography of the human cerebellum using various cognitive tasks and concluded that the left cerebellar hemisphere is responsible for mental manipulation of visual representations. Meanwhile, Maguire *et al*.^56^ conducted a PET study using a virtual environment navigation task, and showed that the cerebellar hemisphere was involved in egocentric aspects of spatial navigation, although cerebellar activation was beyond the scope of their paper. Pine *et al*.^57^ demonstrated that activation in the left cerebellar hemisphere was correlated with memory-guided navigation performance in a virtual environment.

Given the above findings, if functions represented in the left cerebellar hemisphere reflect the peculiarities of car driving, then our current results may imply that disorders accompanied by cerebellar atrophy carry a potential risk of impaired driving. Cerebellar atrophy is observed in various disorders, e.g., attention-deficit/hyperactivity disorder^58^, Alzheimer’s disease^59^, first-episode schizophrenia^60^, and Huntington’s disease^61^. It is particularly noteworthy, from the perspective of traffic safety, that alcoholic patients also present cerebellar atrophy^62^. This enables us to speculate that alcoholic patients may show impeded driving ability, even in a sober state. Furthermore, alcoholic-induced cerebellar atrophy is likely to persist over a long period of time after abstinence^63^. Although an abundance of studies have addressed the influence of alcohol consumption on driving performance^64^, to the best of our knowledge, no studies have investigated driving competence in sober or abstinent alcoholic patients. This may be an important question for future research.

In the current study, we found differences in cerebellar but not cerebral volume between drivers and non-drivers. This does not, however, deny cerebral involvement in car driving. As abundant neuroimaging data have indicated, there seems to be no doubt that various cerebral areas are active during driving a car, depending on task demands and situations^30^. A possible (but unlikely) interpretation of this dissociation is that an average of approximately 8,200 km of annual driving experience may not be enough to induce cerebral morphological changes. However, previous intervention studies do not support the notion that there is less structural plasticity in the cerebrum compared with the cerebellum. For example, Bezzola *et al*.^7^ reported that 40 h of golf practice was associated with gray matter increases in frontal and parietal regions. Kühn *et al*.^10^ demonstrated that 2 months of video game training for less than 1 hour per day induced significant gray matter volume changes in the cerebrum as well as the cerebellum. A more plausible interpretation is that cerebral involvement during everyday driving is relatively non-specific, and may be similar or identical to that associated with other daily activities. Even in the context of traveling by car, according to a previous positron emission tomography study investigating neural correlates of car driving in real traffic^24^, cerebral activation was found comparable between actual driving and passive driving as a passenger, and the cerebellum was found as the only region more active in actual compared with passive driving. This evidence leads us to suppose that morphological changes within driving-related cerebral regions in drivers, if any, can be obscured if non-drivers frequently use a car as a passenger.

Within the limitations of cross-sectional investigation, the current results demonstrate that a few years of car-driving experience in real traffic is associated with greater GM volume in the left cerebellar hemisphere. However, it is not possible to completely rule out hidden factors confounded with car-driving experience. Any activities and experiences concomitant with the selection of the primary transportation mode (i.e., to use or not use a car) are potential confounding factors between drivers and non-drivers. In that respect, the most obvious limitation of this study is that transportation modes in non-drivers were not fully assessed. Accordingly, given the results of the present study, we cannot specify which aspects of car driving are associated with left cerebellar GM increase. In addition, larger cerebellar GM volume might foster more interest in car driving, rather than be a consequence of car-driving experience. To overcome these limitations, it is desirable to conduct a larger sample, randomized longitudinal study that collects detailed information regarding not only frequency and type of usage of cars in drivers but also transportation modes in non-drivers, as well as the concomitant activities and experiences in both groups.

## Methods

### Participants

Seventy-three young adult volunteers (35 males; 38 females) ranging in age from 20 to 23 years participated in this study. All participants were recruited from universities in Nagoya, Japan and the surrounding area. Nearly half of the participants (17 males; 19 females) were licensed drivers with a minimum 1 year of driving experience and an annual mileage at least 5,000 km, and the other half (18 males; 19 females) were non-drivers who either did not have any type of driver’s license, or had no experience driving a car or riding a motorcycle after the acquisition of a driver’s license. Participants were screened such that they were self-reported free of psychiatric and neurological disorders, severe head injuries, and were not currently receiving psychotropic medication. They had normal or corrected-to-normal vision and were right-handed according to the Edinburgh Handedness Inventory^65^. All participants provided written informed consent and were compensated for their time. This study was approved by the ethical committees of Toyota Central R&D Laboratories, Inc and the National Institute of Physiological Sciences and conducted according to the principles of the Declaration of Helsinki. All methods were performed in accordance with the approved guidelines.

### MRI acquisition

High-resolution structural brain images were collected on a Siemens Verio 3 T MRI scanner (Siemens Medical System, Inc., Erlangen, Germany) with a 32-channel head coil, using a three-dimensional T1-weighted magnetization-prepared rapid gradient-echo (MP-RAGE) sequence. The imaging parameters were as follows: repetition time = 1,800 ms; echo time = 2.97 ms; flip angle = 9° field of view = 250 × 250 mm; matrix size = 256 × 256 pixels; slice thickness = 1.0 mm; and 192 contiguous transverse slices.

### MRI preprocessing

After visually inspecting the structural brain images to exclude data with obvious motion artifact or structural abnormalities from subsequent analysis, we processed the data using the VBM8 toolbox (http://dbm.neuro.uni-jena.de/vbm/), which was part of the SPM8 software package (http://www.fil.ion.ucl.ac.uk/spm/) running on Matlab R2015a (Mathworks Inc., Natick, MA). Essentially, VBM8 image preprocessing includes bias correction, tissue classification, and spatial normalization to the Montreal Neurological Institute space with diffeomorphic anatomical registration through exponentiated Lie algebra^66^. We used the default settings for non-linear modulated VBM, with the exception of the space template of affine regularization, for which we selected the International Consortium for Brain Mapping space template for East Asian brains. This preprocessing produced modulated warped GM images with an isotropic voxel resolution of 1.5 × 1.5 × 1.5 mm. Finally, the modulated warped GM images were smoothed with a 6.0 mm full-width at half-maximum isotropic Gaussian kernel.

### Questionnaires

We conducted two questionnaire-based surveys for each participant, completed on the same day as the acquisition of structural brain images. We assessed habitual physical activity using the Japanese version of the IPAQ^67^. We also assessed general personality traits using the Japanese version of the NEO Five-Factor Inventory (NEO-FFI)^68^. The NEO-FFI comprises 60 items and provides scores for personality traits along the following dimensions: openness to experiences, conscientiousness, extraversion, agreeableness, and neuroticism.

### Statistical analysis

To disconfirm potentially confounding differences between drivers and non-drivers, we examined group differences in age and questionnaire scores via unpaired t tests. Likewise, group differences in terms of gender were assessed with a chi-square test. For all tests, the level of statistical significance was set at *P* < 0.05, two-tailed.

We conducted a voxel-wise analysis of the between-group difference in GM volume with a general linear model using SPM8. Age was incorporated into the model as a nuisance covariate. To restrict the search volume for analysis, we applied a study population-specific explicit optimal threshold GM mask, created using the Masking toolbox (http://www0.cs.ucl.ac.uk/staff/g.ridgway/masking/)^69^. The statistical significance level was set at *P* < 0.05 after family-wise error correction for multiple comparisons in the entire volume of analysis. The results were visualized using the MRIcron software package (http://www.mccauslandcenter.sc.edu/mricro/mricron/). Brain regions showing a significant difference were localized using the Anatomy toolbox (http://www.fz-juelich.de/inm/inm-1/DE/Forschung/_docs/SPMAnatomyToolbox/SPMAnatomyToolbox_node.html)^70^.

## Additional Information

**How to cite this article:** Sakai, H. *et al*. Greater cerebellar gray matter volume in car drivers: an exploratory voxel-based morphometry study. *Sci. Rep.* **7**, 46526; doi: 10.1038/srep46526 (2017).

**Publisher's note:** Springer Nature remains neutral with regard to jurisdictional claims in published maps and institutional affiliations.

## Figures and Tables

**Table 1:** Group characteristics.

	Driver (N = 34)	Non-driver (N = 36)
Age (years)^a^	21.5 ± 0.9	21.0 ± 0.8
Sex (male/female)	15/19	17/19
Annual mileage (×10^3^ km)	8.2 ± 5.0	—
Personality trait^b^		
Openness	29.7 ± 5.1	30.7 ± 6.0
Conscientiousness	26.9 ± 7.3	27.3 ± 6.7
Extraversion	31.1 ± 6.1	28.6 ± 6.5
Agreeableness	30.9 ± 6.1	30.7 ± 7.0
Neuroticism	27.1 ± 8.3	27.1 ± 8.9
Physical activity (MET-hours)^c^	95 ± 95	90 ± 78

Note. All values except for sex are given as mean ± SD.

^a^Significant difference between groups (*P* = 0.033).

^b^Assessed with the NEO Five-Factor Inventory^20^.

^c^Assessed with the International Physical Activity Questionnaire^21^.

**Table 2:** Brain regions showing significantly greater gray matter volume in drivers compared with non-drivers.

Region	Peak coordinates			T-score	Extent
	x	y	z		
Cerebellum (crus II)	−39	−61	−44	5.48	30

Note. Peak coordinates are given in the Montreal Neurological Institute space. The statistical significance level was set to a family-wise error corrected *P* < 0.05 for multiple comparisons in the entire volume of analysis.
